# Return to play following spine surgery

**DOI:** 10.3389/fped.2023.1176563

**Published:** 2023-04-17

**Authors:** Tyler A. Tetreault, Sumeet Garg

**Affiliations:** Department of Orthopedics, University of Colorado School of Medicine, Aurora, CO, United States

**Keywords:** scoliosis, return to sports, sports participation, spinal deformity, spine surgery

## Abstract

Return to physical activity is a primary concern for adolescents with idiopathic scoliosis who are indicated for spinal fusion surgery. Preoperative counseling often addresses questions regarding ability to return to sport, postoperative restrictions, time away from play, and the safety of returning to activities. Previous works have shown that flexibility can noticeably decrease after surgery, and that the ability to return to the same level of play may be impacted by the levels of the spine included in the fusion. Equipoise remains on when patients should be allowed to return to non-contact, contact, and collision play; however, there is a trend toward earlier release to activities over the last few decades. Sources agree, though, that returning to play is safe, with rare instances of complications reported for patients with spinal fusion. Here, we review the literature on the function of spinal fusion levels on flexibility and biomechanics, address factors that may influence one's recovery of sports performance, and discuss safety considerations regarding return play following spine surgery.

## Introduction

Though adolescent idiopathic scoliosis (AIS) is a three-dimensional spinal deformity, it is defined as a frontal plane curvature measuring greater than 10 degrees. The primary concern with AIS is that continued deformity progression may put patients at risk for pulmonary dysfunction, chronic back pain, and body dissatisfaction as adults (1). In skeletally immature patients, treatment is indicated for curvatures greater than 20 degrees, as there is a risk for curve progression with continued growth. Bracing remains the gold standard for the nonoperative treatment of idiopathic scoliosis, with Level I evidence that bracing is effective in preventing curve progression to a surgical threshold (2). However, thoracic curves that reach a magnitude greater than 50 degrees and thoracolumbar curvatures greater than 40–45 degrees are indicated for posterior spinal fusion, as these are the curves that are expected to continue to progress after skeletal maturity (3).

While 2%–3% of the population has idiopathic scoliosis, only 0.3%–0.5% will require treatment. Adolescents with curvatures large enough to require treatment are at risk for psychosocial disturbance (4). Conversely, exercise has shown to improve self-esteem and decrease the risk of depression and anxiety in adolescents (5, 6). The impact of spinal fusion on return to activities is a major concern for patients with AIS that requires surgical treatment (7). Sports participation is a common topic of conversation during preoperative consultations and may include questions regarding the ability to and safety of returning to play after surgery.

Understanding the impacts of spinal fusion on ability and timing of return to play aids patients and their families with decision making regarding when to proceed with spinal fusion surgery. This article will review the current literature on postoperative flexibility, the effects of spinal fusion on body mechanics, and the ability to return to sports after spinal fusion. We also address controversies regarding the timing of return to play and factors that may delay sports participation.

## Impact of spinal fusion on body mechanics

The goals of spinal fusion surgery are to prevent further curve progression and correct spinal deformity. To achieve this, the surgeon removes the posterior facet joints to increase flexibility, places fixation into the spine at the desired levels, and uses a sequence of correction maneuvers to improve spinal alignment. Bone graft is then packed around the implants to allow the included vertebrae to fuse together in the corrected position. With this procedure, motion between the included spinal segments is lost. The amount of spinal motion lost is directly related to the number and type of vertebral segments included in the fusion. In a normal spine, the orientation of the facet joints dictates the degree and direction of motion, with the coronally oriented facets in the thoracic spine allowing for greater rotation than flexion/extension, whereas the sagittal orientation of facet joints in the lumbar spine allow for more flexion and lateral bending. The representative range of motion at each motion segment is summarized in Table 1. Understanding the normal biomechanics of the spine allows us to predict what type of spinal motion is lost with spinal fusion.

**Table 1:** Representative values for range of motion of the thoracic and lumbar spine (8).

Motion Segment	Combined flexion/extension (degrees)	One side lateral bending (degrees)	One side axial rotation (degrees)
T1-T6	4	5–6	8–9
T6-T10	5–6	6	4–7
T10-L1	9–12	6–9	2–4
L1-2	12	6	2
L2-3	14	6	2
L3-4	15	8	2
L4-5	16	6	2
L5-S1	17	3	1

Included levels for spinal fusion are determined before surgery based on the curve pattern. The Lenke classification system for AIS helps define which curves are structural and nonstructural and can assist with preoperative level selection (9). Given the role of the lumbar spine with spinal flexion and bending, concerns regarding loss of motion arise as the fusion extends caudally into the lumbar spine. The impact of the chosen lowest instrumented vertebra (LIV) on overall spinal motion after surgery has been well studied and is surveyed below.

For a selected LIV at T11, T12, or L1 there is only a mild decrease in forward flexion compared to preoperative measurements, and there is no significant difference in lumbar mobility for fusions with an LIV between T11 and L1 (10). However, patients with an LIV at L1 or L2 will have a significant decrease in spinal rotation from preoperative levels, and those with an LIV at L3 have significantly less spinal rotation and side bending (11). As LIV moves more distally, there is a continued loss of side bending and forward flexion (12–14). Fan et al. found that for an LIV of L1 or above, patients demonstrated 66 degrees of lumbar flexion, while those fused to L5 had only 23 degrees of lumbar flexion (12). Moreso, patients with a substantial (>40%) reduction from their preoperative mobility have worse quality of life and pain scores after fusion (13, 14).

It is well accepted that spinal fusion with a more caudal LIV will have greater impacts on overall spine range of motion. Given this, investigators have sought to understand these implications on global body mechanics and physical function. In general, overall function seems well preserved in patients with spinal fusion. Gait motion analysis has shown that patients with scoliosis have a decreased range of motion of the pelvis and lumbar spine when walking, both before and after spinal fusion, and that LIV played a limited role in the lack of range of motion (15). That said, in the test called the stop-jump task, used to assess for agility and trunk control, athletes with a history of spinal fusion performed similarly to non-affected controls (16), suggesting that spinal fusion does not significantly impact their ability to participate in sports that require such types of body control. However, it is possible that these in-lab findings are not specific enough to capture functional differences that athletes may notice when returning to a sport that requires excessive bending or twisting, such as dance or gymnastics.

## Recovery of sports performance

An ability to return to their pre-fusion chosen sport is a primary concern for athletes prior to spinal fusion. There is a high probability for return to sports participation at the same level of competition (such as JV, varsity, or club level), with reported rates of return to sport that vary from 59.5% to 96.2% (17–22). While most patients can compete at similar levels and within the same sport, it is important to note that some athletes may choose to switch sports. There is a studied trend away from contact and collision sports after spinal fusion surgery, such as horse riding, ballet, soccer and volleyball, to non-contact and non-collision sports, such as cycling and swimming, at rates as high as 37% (21, 23).

There is no consensus regarding which preoperative or surgical factors are predictive of an ability to return to sports. Early reports on return to sport after spinal fusion suggested that LIV was a significant predictor (17, 18). As LIV moved caudal, Fabricant et al. found a “stepwise decline in percentage of patients returning to athletics at the same or higher level of participation based on distal level of fusion” (17). It is reasonable to think that as LIV becomes more distal and spine motion decreases, return to play will also decrease. However, this finding has not been corroborated in more recent prospective studies, which have shown that LIV is not predictive of return to sport, and that patients with a more distal LIV can return to sport at high rates that are not significantly different than those with a more proximal LIV (22, 24). Various other factors have been proposed as more predictive of rates of return to play. Spine outcomes scores (SRS-22), young age, lower curve magnitude, and Lenke classification have been reported as significant of factors for timing and/or ability to return to sport (17, 21). It is likely that the lack of consensus or consistent findings in return to sports studies is due to the variation in the studies themselves, including: heterogeneity of the subjects (both in age, level of competition, and sport type); study design (study size, predominantly retrospective); variety in outcome measures (return to any sport, change in sport, classification of level of participation); surgical variables (including the extent of correction, restoration of sagittal alignment, or anchor choices); or inability to capture external factors (such as a change in preferences, school or social circumstances that may drive an athlete out of sport).

## Timing to return to play

### When do surgeons release athletes to play? A shifting paradigm

Perhaps the major factor confounding factor in timing of return to sport is when surgeons allow return to activity after surgery. Historically, returning to sports after spinal fusion was not a given. Prior to the 1970s, when spinal fusions were performed without implants, patients were placed in plaster body casts after surgery to maintain their correction. Recovery was a protracted process, and fears of injury with return to sports led most surgeons to restrict these activities. As implants became more widely utilized in the 1970s and 1980s, the decision to allow return to sports was made with consideration of the levels and extent of fusion performed, as well as the desired sport activity and expected level of contact (25).

From the 1990s and onward there was increasing use of more rigid fixation into the spine with pedicle screws. These screws allow for fixation of all three columns of the spine as they extend from the posterior spine, through the pedicles, and into the vertebral bodies. This allows for a very stable construct which typically eliminates the need for bracing after surgery.

In 2002, a survey of Scoliosis Research Society (SRS) surgeons found that factors such as time from surgery, instrumentation type (pedicle screws vs. hooks or bands), LIV, and chosen sport impacted when surgeons allow patients to return to activity (26). By six months after surgery, most surgeons allowed patients to return to gym class and non-contact sports, and at twelve months most allowed for the return to contact sports. However, 11% still forbade contact sports and 20% forbade participation in collision sports. By 2013, perspectives had progressed. A survey of Spine Deformity Study Group (SDSG) surgeons revealed that most surgeons allowed return to running at three months, non-contact and contact sports at six months, and collision sports at twelve months (27).

While there remains no consensus on when to allow patients to return to activity, the pendulum continues to swing. Now, some surgeons release patients to all activities as early as 4–8 weeks after surgery (22). The use of all pedicle screw constructs, decreased surgical time, and low rate of complications with early return to sport are likely the driving factors in these decisions, though there is still no agreement on when to allow return to activity and return to play protocols vary by surgeon.

### When do athletes achieve preoperative levels of competition?

After release to activity, many patients inquire about how long before they will be “back to full strength” and able to compete as they did prior to surgery. An approximate timeline for return to the preoperative level of play allows for families to plan for surgery with adequate time to recover for an important sports season.

The timing of return to the preoperative level of competition appears to be primarily impacted by when patients are released to full activity. For more conservative protocols, where patients are released to non-contact activity at 6–12 weeks, and contact sports at 6–12 months, 51.4% participate in preoperative levels of competition by 6 months, and 88.5% by 12 months (19). Even when preoperative level of competition is achieved, many report a reduced intensity of play or time in sport compared to preoperative levels during the early recovery period (18).

When release to activity after surgery is earlier, return to sport occurs more quickly. Early return to sport protocols may allow patients to return to all activities as tolerated immediately, or starting at 4–8 weeks after surgery (20, 22). At three months, 25–52.6% of patients have returned to their presurgical level of play. By six months, approximately 55%–75% of athletes have achieved this metric, and by twelve months 90%–96% are back to their presurgical levels. With this protocol, sport type is the primary predictor of timing of return to activity. The median return to play for non-contact athletes occurred at 2.2 months vs. 4.7 months for contact athletes (22). No surgical risk factors, including LIV, correlated with a delayed return to sport, though patients reported that back pain and flexibility were the primary barriers in achieving their preoperative level of play.

## Safety of return to play

After surgery, it can take up to 6–12 months for spinal fusion to complete. During this phase, spinal implants maintain alignment of the spine and bear the physiologic stresses from movement. Concerns with early return to sport during this period are primarily due to the risk of implant failure—that excessive forces on the body can cause screw pullout or rod fracture. The risk of pseudarthrosis, or an inability to achieve adequate bony fusion, from excessive motion is also plausible.

In a 2002 survey of SRS surgeons, 19% reported that they had experienced minor complications that they attributed to return to physical activity, such as anchor failure, rod fracture, or pseudarthrosis (26). In more contemporary studies evaluating return to play, however, complications were rare—one instance of set cap dislodgement was noted at 3 years post-op (22) and one patient had failure of their implants when returning to snowboarding before release to activity at only two weeks after surgery (27). Many authors report no early complications related to return to sport (17, 20, 21). However these studies were limited by length of follow-up, which ranged from one (19) to 5.5 years (17). The impact of spinal fusion on long-term sports activity, and whether there is an increased risk for injury to the spine or extremities has not been performed and is an area for future study.

## Discussion

Posterior spinal fusion is a common surgery that can successfully halt progression of idiopathic scoliosis and improve spinal deformity. The primary concerns with spinal fusion are that loss of motion through the involved spinal segments can decrease overall flexibility, impact body mechanics, and potentially put patients at increased risk for injury or complications with return to sport.

Despite some loss of motion following spinal fusion, athletes tend to compensate well. In gait and agility motion analyses, performance is not significantly different from non-affected controls. Analysis of more sport-specific motions necessary for sports that require extreme truncal flexibility is lacking, and there is conflicting evidence over if and how much the unfused spine segments compensate for loss of motion at the levels above (28, 29). Yet, athletes appear able to adapt to these sports as well. Athletes with AIS have shown an ability to return to sports such as golf and gymnastics after spinal fusion (30, 31). Future motion analysis studies assessing the role of LIV and compensatory lumbar hypermobility with sport-specific motions may provide useful information regarding body mechanics for athletes who which to return to sports such as gymnastics or dance that require extreme truncal flexibility.

Most athletes return to their preoperative level of play. Rates of return to play at the same level of competition vary from 59% to 96% (17, 22). There is no consensus on which factors predict difficulty with return to sport. LIV, Lenke classification, curve magnitude, age, and SRS-22 scores have all been proposed. The lack of consensus regarding these findings suggests that external patient factors such as motivation or changes in preference may play an equally significant role in returning to sport as any surgical variable. Athletes report barriers such as back pain, loss of flexibility, deconditioning, and fear of injury as common reasons for difficulty returning to the same level of play (19, 22, 23). These factors may drive some athletes to change sports as well, as trends away from collision and high impact sports towards non-contact sports has been observed at rates as high as 37% (23).

Timing to return to play at a preoperative level of competition is most dependent on surgeon's release to sport rather than any preoperative or surgical variable. Athletes are often ready to return to sport before the surgeon's release, and when an accelerated return to sport protocol is used, athletes can return to sport as early as 3 months after surgery. We support an early release to activity at 4–8 weeks after surgery, as this has allowed adequate time for soft tissues to heal, and modern spinal fusion constructs can withstand the forces associated with sports activity before bony fusion is complete. Allowing athletes to work with their coaches and trainers to determine when to return to competition is valid, as these supervisors have a more complete understanding of the athlete's recovery of on-field performance and the demands of the sport than the surgeon. Establishing a consensus on when return to sport is appropriate would provide valuable guidance to patients and their families. Future prospective studies should closely monitor a large cohort of athletes from many sports disciplines to confirm that early return to sports is safe and effective.
